# Tracheostomy in children is associated with neutrophilic airway inflammation

**DOI:** 10.1136/thorax-2022-219557

**Published:** 2023-02-20

**Authors:** Jason Powell, Steven Powell, Michael W Mather, Lauren Beck, Andrew Nelson, Pawel Palmowski, Andrew Porter, Jonathan Coxhead, Ann Hedley, Jonathan Scott, Anthony J Rostron, Thomas P Hellyer, Fatima Zaidi, Tracey Davey, James P Garnett, Rachel Agbeko, Chris Ward, Christopher J Stewart, Clifford C Taggart, Malcolm Brodlie, A John Simpson

**Affiliations:** 1 Translational and Clinical Research Institute, Newcastle University, Newcastle upon Tyne, UK; 2 Department of Paediatric Otolaryngology, Great North Children’s Hospital, Newcastle upon Tyne, UK; 3 Biosciences Institute, Newcastle University, Newcastle upon Tyne, UK; 4 Department of Applied Science, Northumbria University, Newcastle upon Tyne, UK; 5 Protein and Proteome Facility, Newcastle University, Newcastle upon Tyne, UK; 6 Bioinformatics Support Unit, Newcastle University, Newcastle upon Tyne, UK; 7 Discovery and Translational Science, Metabolon, Morrisville, North Carolina, USA; 8 Electron Microscopy Research Services, Newcastle University, Newcastle upon Tyne, UK; 9 Department of Paediatric Intensive Care, Great North Children’s Hospital, Newcastle upon Tyne, UK; 10 Experimental Medicine, Queen’s University Belfast, Belfast, UK; 11 Department of Paediatric Respiratory Medicine, Great North Children’s Hospital, Newcastle upon Tyne, UK

**Keywords:** Respiratory Infection, Paediatric Lung Disaese, Oxidative Stress, Neutrophil Biology, Lung Proteases, Innate Immunity, Critical Care, Bacterial Infection

## Abstract

**Background:**

Tracheostomies in children are associated with significant morbidity, poor quality of life, excess healthcare costs and excess mortality. The underlying mechanisms facilitating adverse respiratory outcomes in tracheostomised children are poorly understood. We aimed to characterise airway host defence in tracheostomised children using serial molecular analyses.

**Methods:**

Tracheal aspirates, tracheal cytology brushings and nasal swabs were prospectively collected from children with a tracheostomy and controls. Transcriptomic, proteomic and metabolomic methods were applied to characterise the impact of tracheostomy on host immune response and the airway microbiome.

**Results:**

Children followed up serially from the time of tracheostomy up to 3 months postprocedure (n=9) were studied. A further cohort of children with a long-term tracheostomy were also enrolled (n=24). Controls (n=13) comprised children without a tracheostomy undergoing bronchoscopy. Long-term tracheostomy was associated with airway neutrophilic inflammation, superoxide production and evidence of proteolysis when compared with controls. Reduced airway microbial diversity was established pre-tracheostomy and sustained thereafter.

**Conclusions:**

Long-term childhood tracheostomy is associated with a inflammatory tracheal phenotype characterised by neutrophilic inflammation and the ongoing presence of potential respiratory pathogens. These findings suggest neutrophil recruitment and activation as potential exploratory targets in seeking to prevent recurrent airway complications in this vulnerable group of patients.

**WHAT IS ALREADY KNOWN ON THIS TOPIC**

- Paediatric tracheostomy is associated with significant morbidity and mortality.
- Respiratory complications result in poor quality of life for children and their carers, and substantial healthcare costs.

**WHAT THIS STUDY ADDS**

- The study shows for the first time that, in children, long-term tracheostomy is associated with neutrophilic inflammation, active proteolysis, sustained superoxide production and airway dysbiosis.
- These data suggest an association between airway host defence dysregulation, microbiological changes and airway infections.

**HOW THIS STUDY MIGHT AFFECT RESEARCH, PRACTICE OR POLICY**

- Modulation of neutrophilic inflammation appears to be a potential target for prevention of airway inflammation and infections in this cohort and should be the focus of future clinical trials.

## Introduction

Tracheostomy in children is performed to facilitate long-term ventilation, assist weaning or overcome airway obstruction.^1^ Most paediatric tracheostomies are performed in infancy, with children remaining cannulated for many years, and often life-long.^1 2^ Tracheostomy has multiple benefits over other methods of intubation and ventilation. However, in children it is frequently associated with ongoing respiratory complications, accounting for up to half of all hospital readmissions.^2–5^ Frequent hospitalisation is associated with significant healthcare costs, and a negative impact on quality of life for children and their carers.^2–7^ Mortality rates in tracheostomised children are in the region of 10%–30% with later respiratory complications the predominant causes of death.^2–9^ Paediatric tracheostomy is commonly associated with antibiotic-resistant pathogens.^10 11^ Children with tracheostomies are regularly prescribed antibiotics for respiratory complications, however, increasing evidence demonstrates the detrimental long-term health effects of frequent antibiotic use in childhood.^12^

Introduction of the tracheal tube is likely a key contributing factor to respiratory complications, bypassing the protective function of the nose, pharynx and larynx. Post-tracheostomy, airway host defence mechanisms protect against the development of bronchitis and pneumonia. In infants and young children, these defences are less mature than in adults, increasing the risk of infection.^13^ It has previously been demonstrated that the alveolar airspace in tracheostomised children is characterised by increased total protein and an increase of both relative percentages and absolute numbers of neutrophils.^14^

Remarkably, little else is known about the effect of tracheostomy on host defence disruption in children. Identification of host factors associated with tracheostomy in children would likely suggest targets for future clinical trials seeking to prevent respiratory complications in this vulnerable patient group. The aims of this study were therefore to perform a novel, serial characterisation of the host and microbe environment in tracheostomised children, through a systematic multiomic evaluation of the tracheal airway.

## Methods

### Patients and controls

**Figure 1:**
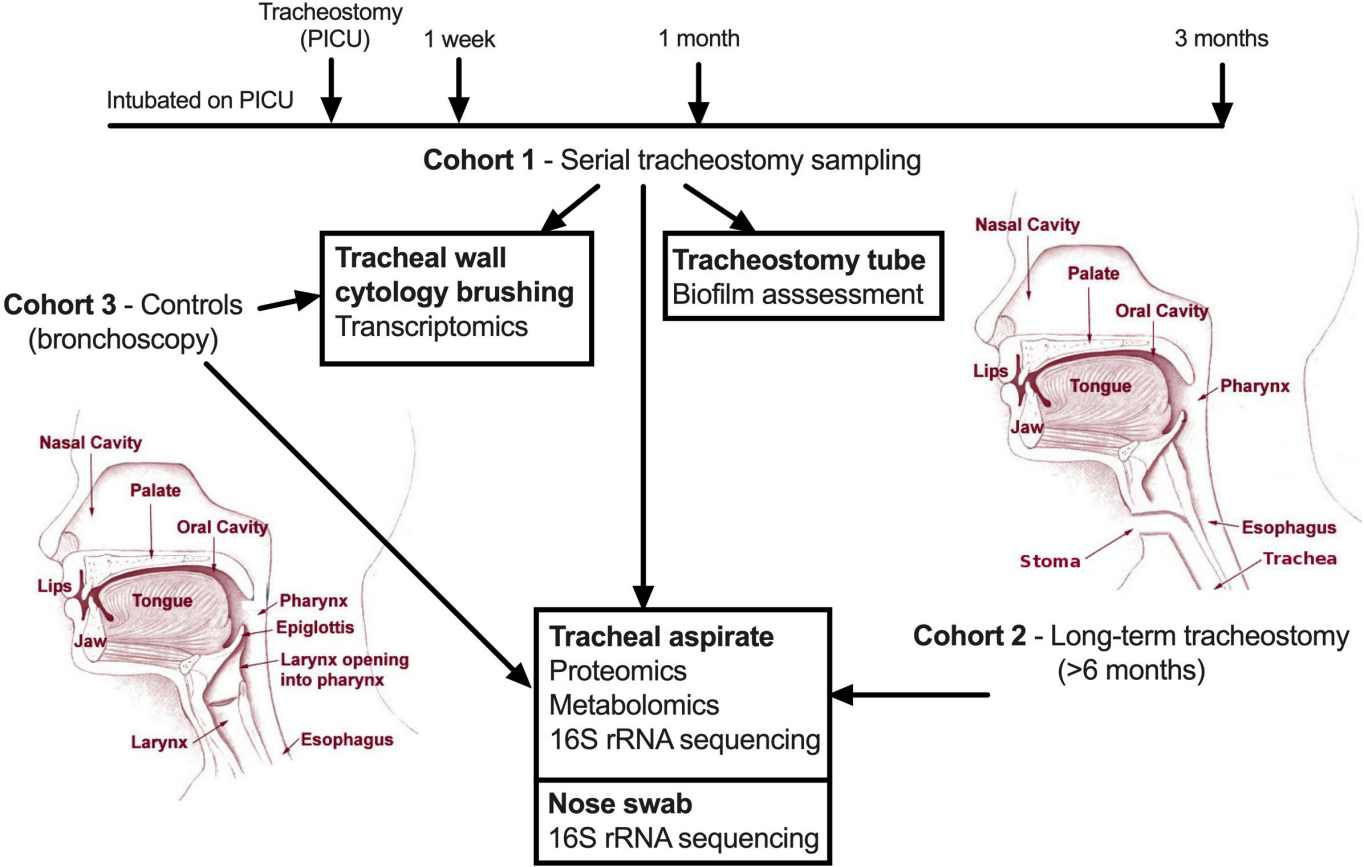
Schematic depiction of the sampling protocol and subsequent analysis of samples generated. PICU, paediatric intensive care unit.

Tracheostomy cohorts included children enrolled at the time of tracheostomy and followed up at 1 week, 1 month and 3 months post-tracheostomy (serial cohort) or those with an established tracheostomy which had been in place for over 6 months (long-term cohort), who were sampled at a single time point. Controls (sampled at a single time point) were children without a tracheostomy undergoing elective bronchoscopy under general anaesthesia for investigation of structural airway problems, such as obstructive sleep apnoea. Both tracheostomy cohorts and the control group had collection of a tracheal aspirate and nasal swab (nylon Sterilin flocked swab, Fisher Scientific, Massachusetts, USA). Controls and the serial tracheostomy cohort also had tracheal wall brushings collected using a sheathed cytology brush (BC-202D-5010, Olympus, Tokyo, Japan). Further details relating to groups and sampling are in online supplemental file 1 and figure 1. Informed, written consent was obtained from a main carer in all groups.

10.1136/thorax-2022-219557.supp1 Supplementary data

### RNA sequencing

RNA was extracted from brush heads using the RNeasy mini kit (Qiagen, Hilden, Germany) and stranded total RNA sequencing performed (details are contained in online supplemental file 1).

### Proteome in tracheal aspirates

Proteome analysis was performed using liquid chromatography-mass spectrometry of tracheal aspirates. Human proteins were identified using MaxQant (details are contained in online supplemental file 1).^15^ The differentially abundant proteins generated were uploaded into Ingenuity Pathway Analysis (IPA) software. An activation Z score (where positive predicts activation and negative inhibition of a pathway) was generated by applying the protein-associated genes and the strength of their known associations with biological networks (details are contained in online supplemental file 1).^16^

### Metabolome in tracheal aspirates

Metabolome testing of tracheal aspirates was performed by Metabolon (Morrisville, North Carolina, USA). Metabolome profiling used ultra-high-performance liquid chromatography-tandem mass spectrometry. Details of the sample preparation, metabolome profiling, identification of compounds and quality control are in online supplemental file 1.^17^

### 16S rRNA sequencing in nasal swabs and tracheal aspirates

Microbial DNA was extracted from the nasal swabs and tracheal aspirates using the PowerSoil DNA Isolation Kit (Qiagen, Hilden, Germany) as described previously.^18^ 16S rRNA gene sequencing was performed by NU-OMICS (details are contained in online supplemental file 1).^19^

### Biofilm assessment

Tracheostomy tubes were collected at the first tube change (approximately 1 week postprocedure) and the inner lumen assessed for biofilm formation using scanning electron microscopy (details are contained in online supplemental file 1).

### Statistical analysis

Statistical tests are described with the associated analysis in the online supplemental methods. P value <0.05 was considered statistically significant with the false discovery rate algorithm used to adjust for multiple comparisons.^20^ Figures were generated from the associated analysis software as described in the online supplemental methods or in GraphPad V.9.3.1. Multi-omic data integration was carried out on samples assayed multiple times using different platforms. Multiple Co-Inertia Analysis (MCIA) was performed using the omicade4 R package V.1.32.0 to integrate the datasets.^21^

## Results

### Patients and controls

Thirty-three tracheostomised patients and 13 controls were recruited. Nine patients were recruited prior to their tracheostomy procedure and followed serially for 3 months (serial cohort). All patients in the serial cohort had been intubated and mechanically ventilated on the intensive care unit for a median of 24 days (IQR, 11–36 days; range 7–142 days) prior to tracheostomy. The remaining 24 patients (long-term cohort) had long-term tracheostomies that had been in place for a median of 31 months (IQR, 14–44 months; range, 7–140 months). In the serial cohort, two patients died of cardiac complications related to pre-existing co-morbidities, and one was decannulated, during follow-up. Samples collected up to that point were included in the analysis. Clinical and demographic data are described in table 1. Further details are in online supplemental table S1–S3.

**Table 1:** Demographic and clinical data for patient and control groups

	Serial tracheostomy (n=9)	Long-term tracheostomy (n=24)	Bronchoscopy controls (n=13)
Median age at enrolment, months (IQR)	4 (2–10)	35 (14–54)	28 (13–40)
Percentage male (%)	67	71	53
Percentage premature <38 weeks (%)	56	54	31
Median weight in kilograms at enrolment (IQR)	5 (4–7)	13 (10–16)	13 (9–15)
Caesarean section delivery (%)	33	29	46
Non-oral feeding route (%)	100	63	15
Antibiotics prescribed in the year prior to enrolment (%)	100	83	31

### Serial tracheostomy cohort

**Figure 2:**
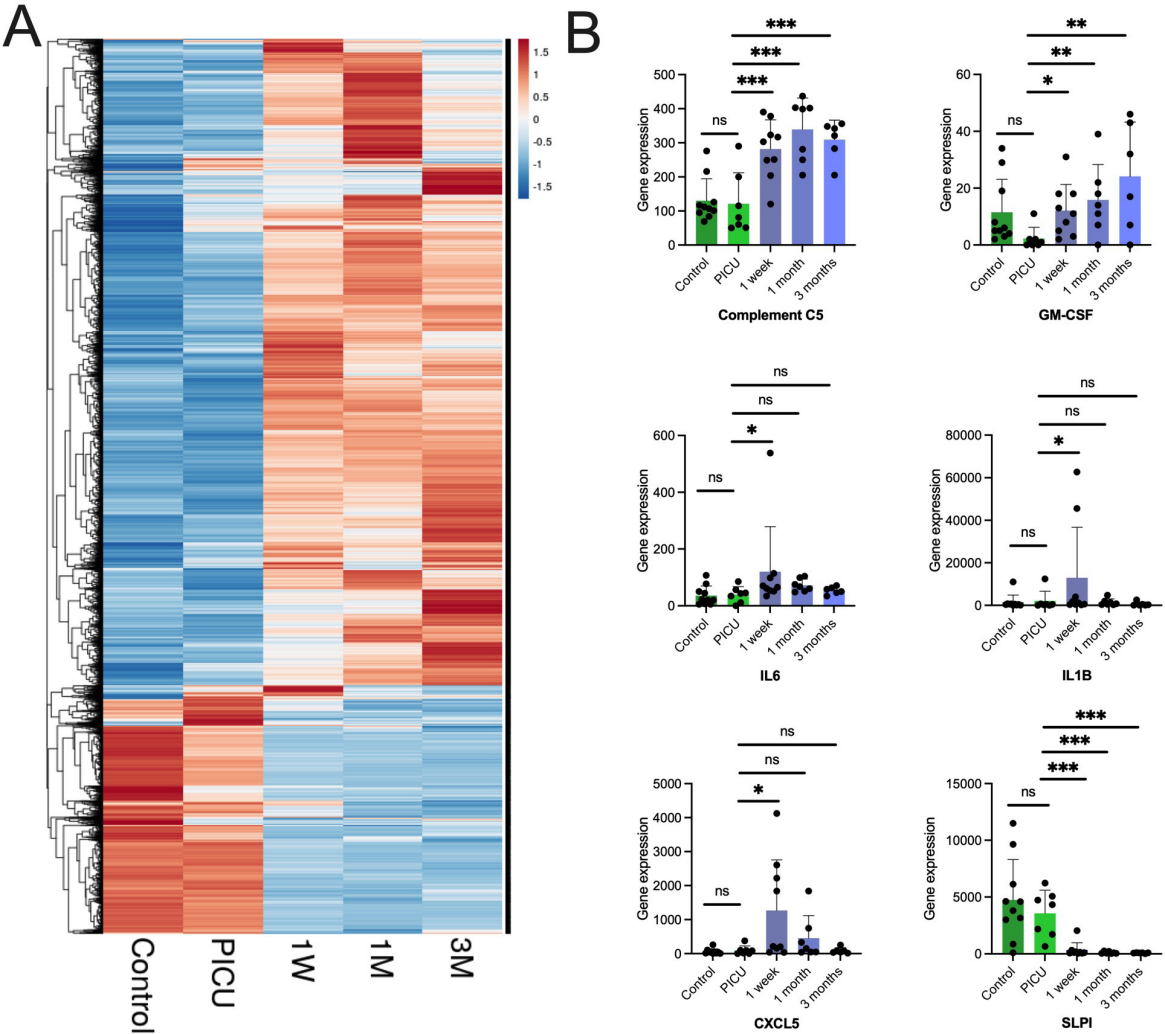
Tracheal wall brushings post-tracheostomy demonstrate early and persistent transcriptomic changes associated with neutrophilic inflammation. (A) Hierarchical clustering of tracheal brushing samples by gene expression from RNA-seq where each column represents a sample group and each row a gene. The colour indicates the z-score by gene, on read counts, normalised for sequencing depth. PICU indicates sampling at the time of tracheostomy, and subsequent time points are post-tracheostomy, control data are also shown. (B) Individual serial gene expression profiles in tracheal brushing samples for chemokines, cytokines and complement factors. Columns indicate means and error bar the SD. PICU indicates the time of tracheostomy, and is presented alongside post-tracheostomy and control data. Statistical analysis was in DESeq2 using a modified negative binomial Wald test, *p<0.05, **p<0.01, ***p<0.001 (controls n=10, serial; PICU n=7, 1 week n=9, 1 month n=7, 3 months n=6). PICU, paediatric intensive care unit (time of tracheostomy); SLPI, secretory leucocyte protease inhibitor; 1W, 1 week post tracheostomy; 1M, 1 month post-tracheostomy; 3M, 3 months post-tracheostomy.

Tracheal wall cytology brushing samples derived post-tracheostomy demonstrated a distinct transcriptome compared with samples at the time of tracheostomy, and samples from controls (figure 2A). Examining expression of specific genes post-tracheostomy, compared with the time of tracheostomy, a statistically significant increase in the expression of specific chemokines, cytokines and complement factors implicated in neutrophil recruitment was observed (figure 2B). This included a non-sustained increase in IL-6, IL-1β and CXCL5 at 1 week post-tracheostomy, and a sustained increase in GM-CSF and complement C5 (which is cleaved into its active forms C5a (a potent neutrophil chemoattractant) and C5b), at 1 week, 1 month and 3 months post-tracheostomy.

There was also a statistically significant reduction in the expression of secretory leucocyte protease inhibitor, a key airway antiprotease, post-tracheostomy.^22^ In parallel, a proteomic assessment of tracheal aspirates demonstrated a trend towards an increased abundance of neutrophil proteases post-tracheostomy (online supplemental figure S1). Metabolomic analysis also detected a significant increase in dipeptide species from 1 week post-tracheostomy, compared with the time of tracheostomy, indicative of new active proteolysis (online supplemental table S4).

**Figure 3:**
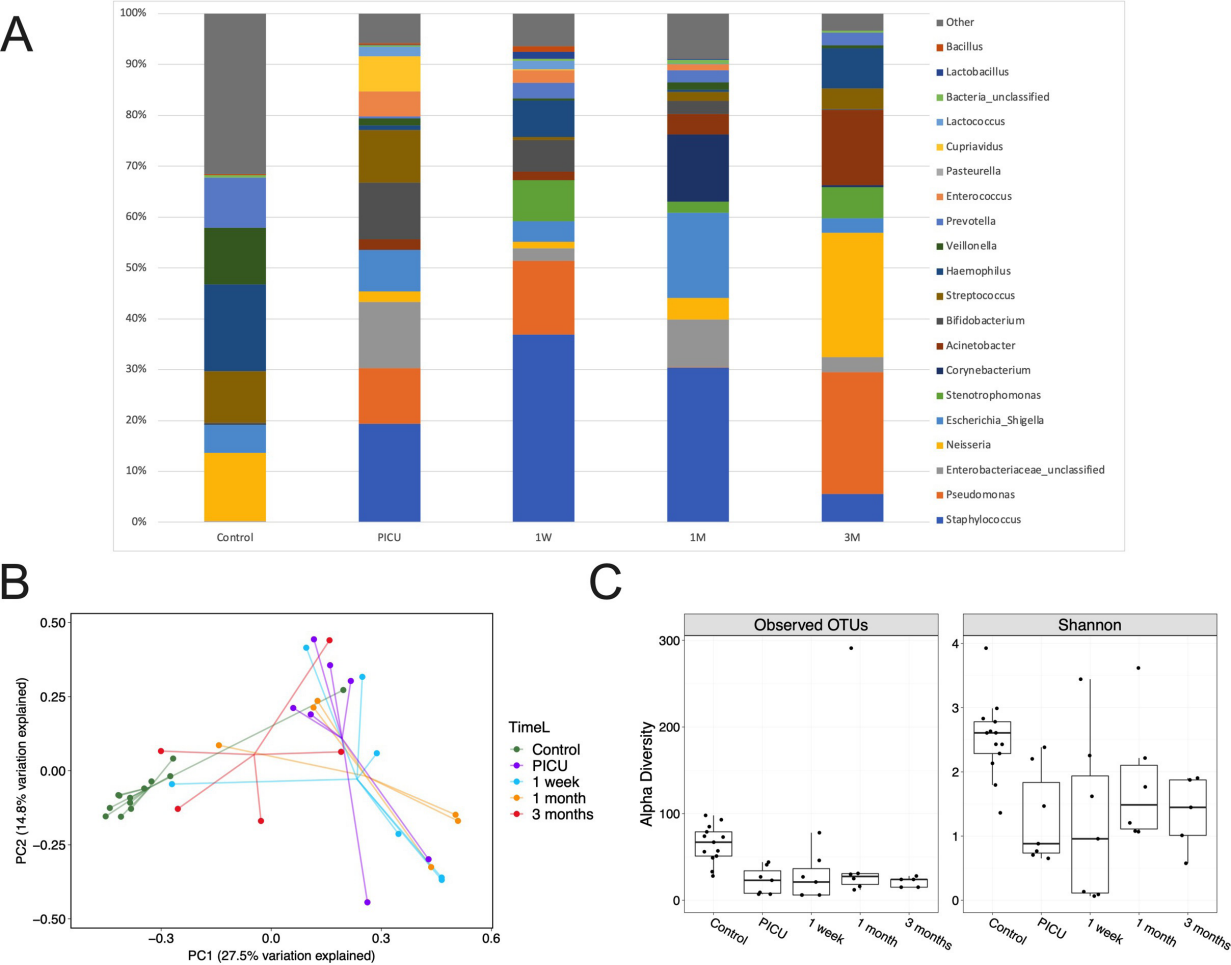
Tracheostomy is associated with an ongoing increased burden of potentially pathogenic organisms and reduced microbiological diversity, however these changes are acquired prior to tracheostomy. (A) Serial comparisons of 16S rRNA sequencing of tracheal aspirates demonstrating relative bacterial abundance from the time of tracheostomy (PICU) to 3 months postprocedure. Control samples are shown for comparison. Top 20 most abundant genera in the serial tracheostomy samples are demonstrated. (B) Principal component analysis comparing tracheal aspirate microbiomes. (C) Operational taxonomic units (OTUs) (p<0.01) and Shannon alpha diversity (p<0.01) are demonstrated for tracheal aspirates. Box limits show the upper and lower quartiles, horizontal lines indicate the median, whiskers represent the range. Controls n=13, serial; PICU n=7, 1 week n=7, 1 month n=8, 3 months n=5. PICU, paediatric intensive care unit (time of tracheostomy).

We postulated that these host changes post-tracheostomy would be associated with alterations in the microbial ecology of the airways. Using 16S rRNA sequencing of tracheal aspirates no substantial change in the composition or diversity of organisms from the time of tracheostomy to postprocedure was demonstrated (figure 3A–C). However, the diversity and relative abundance of organisms at the time of and post-tracheostomy were considerably different from control samples (figure 3A–C). All tracheostomy patients were already intubated and mechanically ventilated on the intensive care unit prior to tracheostomy. Within 1 week, tracheostomy tubes showed evidence of biofilm formation and bacterial colonisation (online supplemental figure S2).

### Long-term tracheostomy cohort

**Figure 4:**
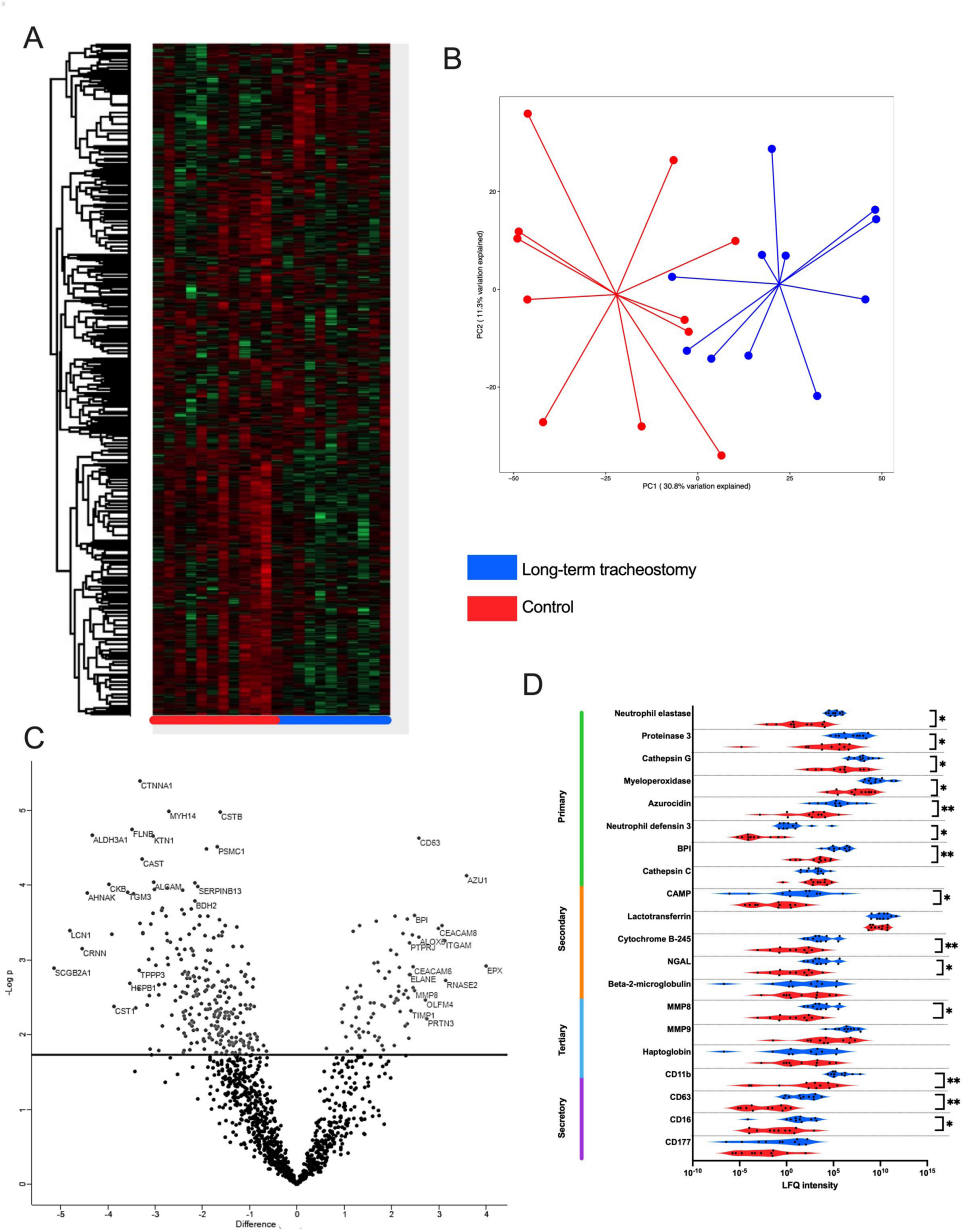
Long-term tracheostomy is associated with evidence of neutrophilic inflammation in airway samples. (A) Hierarchical clustering of tracheal aspirate samples by protein intensity—rows represent individual proteins and columns individual patients by sample groups. Red: control; blue: long-term tracheostomised patients. (B) Principal component analysis comparing proteomic findings from tracheal aspirates of long-term tracheostomised patients to controls. (C) Volcano plot showing differentially abundant proteins between the tracheal aspirates from long-term tracheostomised patients and controls. The line indicates the false discovery rate-adjusted p<0.05 cut off. Protein-associated gene symbols are demonstrated for the most significantly differing proteins. (D) Violin plot showing abundance of detectable neutrophil granule-related proteins grouped by granule type. Statistical analysis was by Welch’s two-sample t-test, *p<0.05, **p<0.01, long-term tracheostomy n=11, control n=11. LFQ, label-free quantification; NGAL, neutrophil gelatinase-associated lipocalin; BPI, bactericidal permeability-increasing protein; CAMP, cathelicidin antimicrobial peptide; MMP8, matrix metalloproteinase 8; MMP9, matrix metalloprotease 9; TIMP1, tissue inhibitor of matrix metalloproteinase 1; PRTN3, proteinase 3; CEACAM8, carcinoembryonic antigen-related cell adhesion molecule 8/CD66b; AZU1, azurocidin 1; ITGAM, integrin subunit alpha M/CD11b.

**Figure 5:**
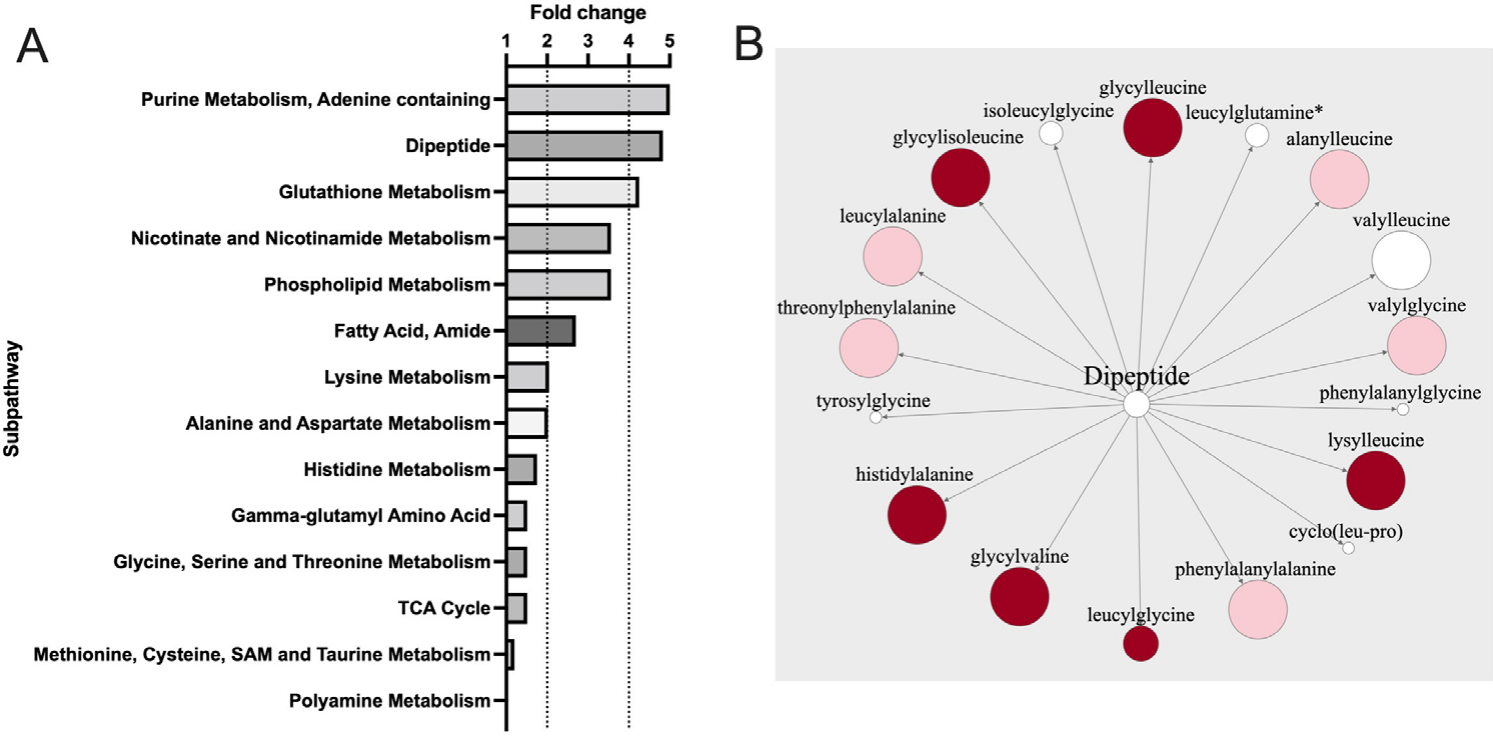
Tracheal aspirates from long-term tracheostomised patients are associated with active proteolysis. (A) Comparative fold-change (positive and negative) associated with metabolomic sub-pathways between patients with long-term tracheostomised patients and controls. (B) Dipeptide metabolite pathway, the size of each circle indicates the relative fold-change in patients with long-term tracheostomised patients compared with controls, dark red indicates p<0.05, lighter red indicates p<0.1, white indicates non-significant changes. Statistical analysis was by Welch’s two-sample t-test, long-term tracheostomy n=18, control n=12.

We aimed to evaluate the airway effects of tracheostomy further in a larger cohort of long-term tracheostomised patients. Tracheal aspirates demonstrated a distinct proteome compared with control aspirates (figure 4A,B). A large proportion of the significantly more abundant proteins identified in long-term tracheostomised patients compared with controls related to neutrophil degranulation (figure 4C). Examining abundance of various neutrophil granule proteins significantly more proteins associated with primary, secondary and tertiary neutrophil granules were identified (figure 4D). This was supported by IPA analysis of the tracheal proteome, where the most significantly activated biological networks in tracheostomised patients compared with controls were related to neutrophil degranulation (activation, z-score 2.59, p<0.001). In keeping with findings in the serial cohort, metabolomic analysis also identified an excess of a range of dipeptides in the airway of patients with long-term tracheostomies (figure 5A,B), indicative of proteolytic activity.

Excess neutrophil degranulation is associated with an oxidative stress response which can be measured metabolically.^23^ A significant reduction in glutathione (fold change 0.36, p<0.05) and elevation in methionine sulfoxide (fold change 29.86, p<0.05), a product of methionine oxidation, was found in the airways of patients with long-term tracheostomies, compared with controls. Collectively these data suggest differences in redox homeostasis and a greater oxidative stress response in long-term tracheostomised patients compared with controls.

**Figure 6:**
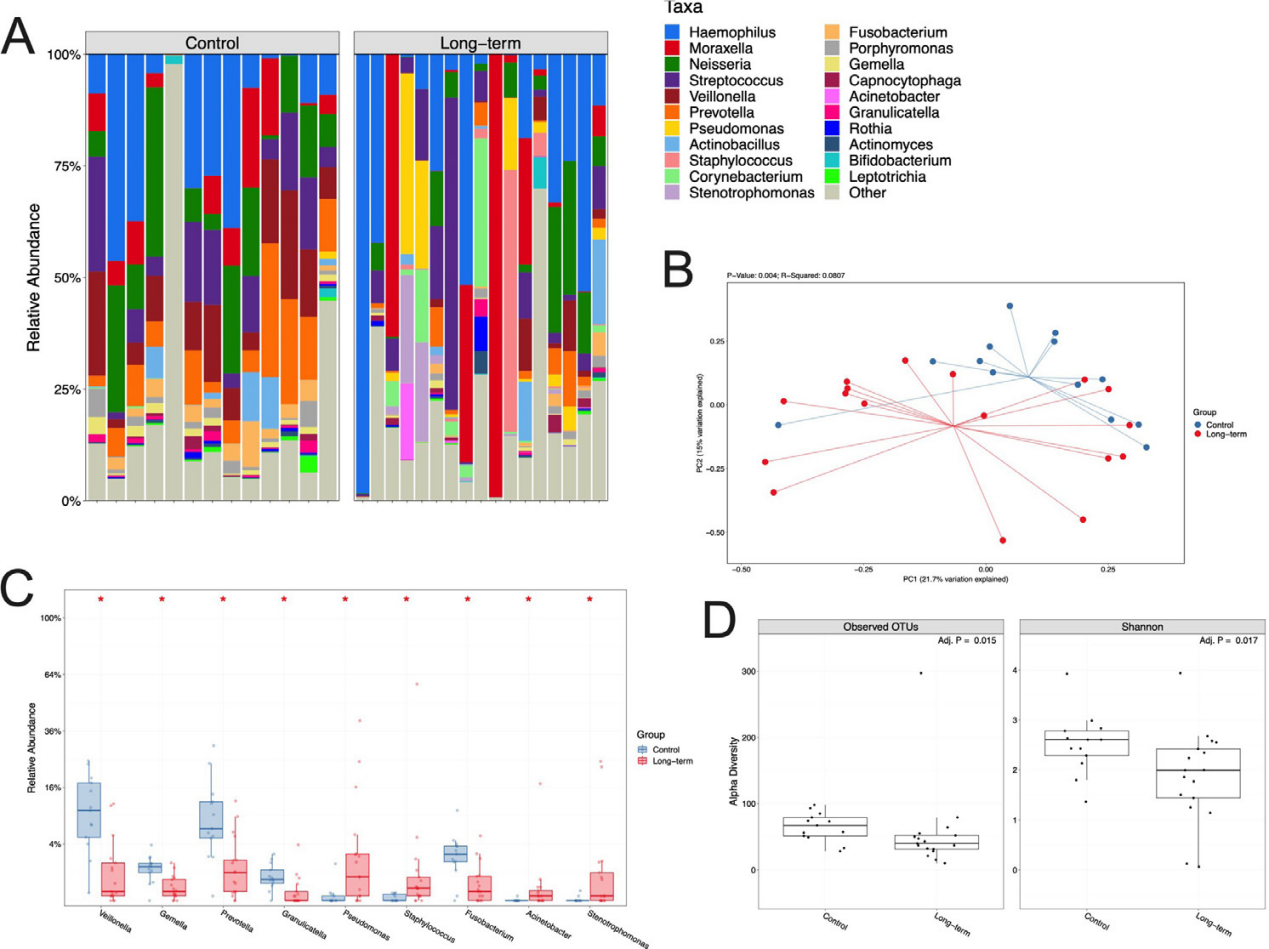
Tracheal aspirates from long-term tracheostomised patients are characterised by reduced microbiological diversity and emergence of potential respiratory pathogens. (A) 16S rRNA sequencing demonstrating hierarchical clustering of tracheal aspirate samples by bacterial relative abundance expressed as observed operational taxonomic units (OTU) levels, where each column represents a patient’s sample. (B) Principal component analysis comparing tracheal microbiomes in long-term tracheostomised patients and controls. (C) Statistically significant differences in bacterial abundance in tracheal aspirates. Box limits represent the upper and lower quartiles, horizontal lines the median, and whiskers the range. (D) OTUs and Shannon alpha diversity for tracheal aspirates. Mann–Whitney U test, *p<0.05. Long-term tracheostomy n=17, controls n=13.

We investigated the microbiological changes associated with a long-term tracheostomy, compared with controls. A distinct microbiome profile was demonstrated in tracheostomised patients, with healthcare-associated respiratory pathogens more frequently identified (eg, *Pseudomonas*, *Staphylococcus*) (figure 6A–C). The richness and Shannon diversity in the tracheal aspirates of long-term tracheostomised patients were significantly lower compared with controls (figure 6D). Control samples tended to contain more commensals, in larger numbers, than samples from tracheostomised patients (figure 6A,C). There was no overall significant difference in the diversity or relative abundance of organisms identified in nasal samples obtained from long-term tracheostomised patients and controls (online supplemental figure S3).

**Figure 7:**
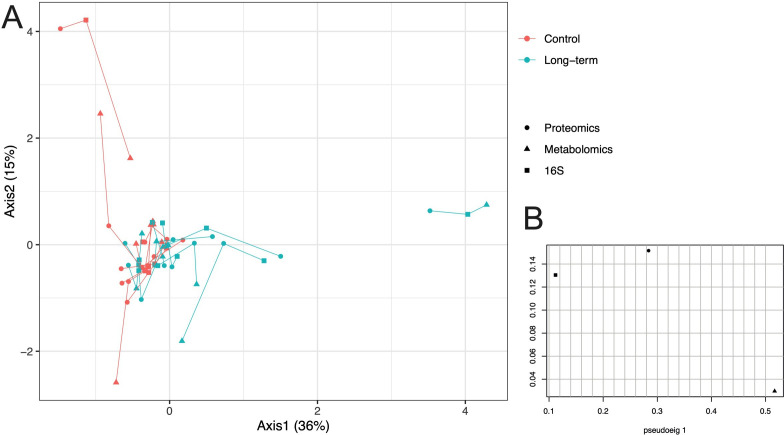
Integration of proteomic, metabolomic and 16S rRNA datasets from tracheal aspirates using multiple co-inertia analysis (MCIA) demonstrates multi-omic variance between long-term tracheostomised patients and controls, but correlation within individuals’ datasets. (A) In the MCIA, lines connect 16S (square), proteomic (circle) and metabolomic (triangle) data for each individual tracheal aspirate sample, from control and long-term tracheostomised patients, which have been projected into the same dimensional space. Correlation between datasets from the same sample are demonstrated by shorter lines. Tracheostomy patient data are in teal, controls in red. (B) Pseudo-eigenvalues of all datasets, indicating how much variance is contributed by the proteome, metabolome or 16S datasets. Long-term tracheotomy n=11, control n=10.

MCIA was used to integrate the three datasets (proteome, metabolome and 16S rRNA sequencing) and visualise any relationships between them. MCIA was performed on tracheal aspirate samples from control and long-term tracheostomised patients, which were projected into the same dimensional space (figure 7). This analysis corroborates analysis on the individual datasets, showing that tracheal aspirate samples from long-term tracheostomised patients had different proteome, metabolome and 16S rRNA sequencing profiles compared with controls (figure 7). Furthermore, this analysis demonstrated that the three datasets were well correlated within patients, as indicated by the short lines connecting individual patients (figure 7).

## Discussion

Our data suggest, for the first time, that tracheostomy in children is associated with airway neutrophilic inflammation and ROS generation. The inference is that the presence of a tracheostomy stoma and tube may drive generation of neutrophil-recruiting cytokines, resulting in ongoing neutrophilic recruitment and inflammation, and leading to sustained release of neutrophil proteases and ROS. These may promote local tracheal inflammation and further neutrophil recruitment, potentially establishing a vicious cycle of chronic inflammation. Persistent inflammation can cause changes in host defence function and subsequent increased risk of infection.^24 25^ Neutrophil recruitment and/or the activation status of airway neutrophils therefore potentially represent targets worthy of exploration in clinical trials seeking to mitigate the adverse effects associated with tracheostomies in children.

Multiple strands of evidence support a pivotal role for the neutrophil in the airway inflammation associated with tracheostomy—IL-6, IL-1β, CXCL5, GM-CSF and activated C5 are all associated with neutrophil recruitment,^26^ and human neutrophil elastase, cathepsin G and proteinase 3 are archetypal neutrophil serine proteases. Neutrophil protease-mediated damage is known to promote tissue degradation, in turn promoting further neutrophil recruitment.^27–29^ Our metabolic data extend these findings by suggesting that ROS generation and increased oxidative stress have functional consequences in the tracheostomised airway, for example, through depletion of glutathione.^30 31^ Oxidative stress occurs when there is an imbalance between the production of free radicals or ROS and the ability of innate antioxidant defences to detoxify these reactive intermediates or repair the resulting damage.^32^ Relatively unopposed oxidative stress can result in damage to proteins, lipids and DNA.^32^ Glutathione plays an important role in antioxidant defence, redox-homeostasis and protein folding.^33 34^ Furthermore, the observed increase in airway methionine sulfoxide, a product of methionine oxidation, is consistent with an oxidising environment.^35 36^

Tracheal aspirates from patients with long-term tracheostomies showed reduced microbial diversity and increased abundance of organisms associated with healthcare-associated respiratory infections, such as *Pseudomonas*. While this has been demonstrated to a limited degree by genomic and non-genomic methods in long-term tracheostomised children previously, we were able to demonstrate for the first time in our serial cohort that the dysbiosis predates the tracheostomy tube placement.^1 10 11 37 38^ This supports previous microbiome research in children demonstrating changes in the airway microbiome with endotracheal intubation and ventilation.^39^ Nevertheless, previous study showed rapid resolution following extubation, with improvements in diversity and reduction in the pathogenic bacterial load.^39^ Therefore, it is likely that the persistent local inflammatory environment caused by the tracheostomy tube prevents restoration of a healthy microbiome. There was no significant difference in the diversity or abundance of organisms identified in the nose, potentially indicating that the observed changes in the trachea related to the tracheostomy rather than to systemic antibiotic use, which was high in the tracheostomy cohorts, compared with the controls.

Limitations of this study include the single site and low patient numbers in our cohorts, however, these children are extremely difficult to recruit, as exemplified by the general lack of data in tracheostomised children. We did, however, apply a comprehensive range of scientific methods to characterise the samples deeply and our findings were consistent. Second, confounding factors such as differing rates of antibiotic use, history of aspiration, age and sex could have explained some of the observed differences between the tracheostomised and control cohorts. Obtaining airway samples for control data is notoriously difficult and controlling for every clinical feature is challenging—the control group was therefore a convenience sample as it was not feasible to match demographic or clinical variables between the tracheostomy groups and controls. Third, we were unable to perform corresponding differential cytological assessment of relative or absolute cell numbers, to corroborate transcriptomic and proteomic evidence of increased neutrophil activity in tracheostomised airways, compared with controls. However, Griese *et al* 14 previously demonstrated an increase in the relative percentages and absolute number of neutrophils, a decreased fraction of macrophages, and increased total protein in the peripheral airspace of children with a tracheostomy, compared with controls. Fourth, transcriptomic analysis was limited by using total RNA sequencing, opposed to single-cell technologies, which would have allowed investigation of cell specific transcriptomic differences. Finally, we cannot be certain that the multiple statistically significant observations equate to clinical significance, though the consistent changes associated with neutrophil dysfunction, the association between tissue neutrophil activity and pathology in multiple other diseases, and the biological plausibility associated with the observed changes may increase confidence in the findings.

In conclusion, tracheostomies in children are associated with an inflammatory tracheal phenotype characterised by neutrophilic inflammation and ongoing presence of potential respiratory pathogens. Our data suggest effective and safe modulation of these processes may represent attractive strategies to investigate in clinical trials, with the ultimate aim of improving paediatric tracheostomy outcomes.

## Data Availability

Data are available upon reasonable request.
