# Supplementary material for: Tracheostomy in children is associated with neutrophilic airway inflammation
Source: Thorax. 2023 Feb 20;78(10):1019–27. doi: 10.1136/thorax-2022-219557 (PMC10511973; doi:10.1136/thorax-2022-219557)
Supplement: Supplementary data [file thorax-2022-219557supp001.pdf]

Full title: Tracheostomy in children is associated with neutrophilic airway inflammation.

Authors: Jason Powell[1, 2], Steven Powell[2], Michael W Mather[3], Lauren Beck[1], Andrew Nelson[4], Pawel Palmowski[5], Andrew Porter[5], Jonathan Coxhead[3], Ann Hedley[6], Jonathan Scott[1], Anthony J Rostron[1], Thomas P Hellyer[1], Fatima Zaidi[7], Tracey Davey[8], James P Garnett[1], Rachel Agbeko[1, 9], Chris Ward[1], Christopher J Stewart[1], Clifford C Taggart[10], Malcolm Brodlie*[1, 11], A John Simpson*[1]

SUPPLEMENTARY METHODS

Patients and volunteers

Tracheostomy cohorts included children enrolled at the time of tracheostomy and followed for up to 3 months (serial cohort). Samples were collected at the time of tracheostomy and subsequently at approximately one week, one month and three months post-procedure. A second cohort comprised children with an established tracheostomy in place for over 6 months (long-term cohort). Exclusion criteria for both tracheostomy cohorts comprised; age >15 years or a diagnosis of hereditary disorder associated with recurrent airway infections, such as cystic fibrosis or primary immunodeficiency. Controls were a convenience sample of children recruited from elective operating lists of patients undergoing airway examination under general anesthesia for investigation of structural (non-infective) airway problems. Exclusion criteria comprised; age >15 years or a diagnosis of a hereditary disorder associated with recurrent airway infections.

Collection of tracheal secretions, airway swabs and tracheal brushings

Eligible patients had tracheal secretions collected by an experienced operator. Briefly, in non-tracheostomised patients secretions were suctioned under direct vision from the trachea via a bronchoscope or laryngoscope. A nasal swab was also collected for microbiome analysis. It was not possible to collect every sample at each visit due to patient compliance with sampling (for example cytology brushings in awake participants) or failure to provide samples (for example no aspiration within the allocated timeframe). All samples were transported to the laboratory fresh and immediately frozen at -80°C until further processing.

RNA sequencing

RNA was extracted from tracheal wall brushings using the RNeasy mini kit (Qiagen, Hilden, Germany) and stranded total RNA sequencing libraries were prepared using the SMART-Seq Stranded Kit (Takara Bio, CA) and SMARTer RNA Unique Dual Index Kit (Takara Bio, CA) following the manufacturer's protocol. Libraries were quantified using a TapeStation 4200 (Agilent Technologies, CA) and Qubit 4 (Thermo Fisher, MA) and equimolar samples pooled. The pooled library was sequenced at ~80 million (2 x 100 base pair) reads per sample on a NovaSeq 6000 using an S2 200 cycle flow cell (Illumina, CA). Data for individual samples were demultiplexed into separate FASTQ files using Illumina's bcl2fastq software (version 2.20.0.422) and quality checks on the raw data were performed using FastQC (version 0.11.50) and Fastq Screen (version 0.14.1). Alignment of the RNA-seq paired-end reads was to the GRCh38.104 version of the human genome, and annotation performed using Hisat2 (version 2.2.1). Expression levels were determined and statistically analysed by a workflow combining HTSeq (version 0.6.1), the R environment, utilising packages from the Bioconductor data analysis suite and differential gene expression analysis based on the negative binomial distribution using the DESeq2 package. All FDRs were calculated using the DESeq2 package default method. Non-protein-coding genes were excluded from downstream analysis. Further data analysis and visualisation used R (environment version 4.0.3) and Bioconductor packages.

Proteome testing

Tracheal aspirate samples were mixed with sodium dodecyl sulfate solution, heated at 95°C and sonicated to lyse cells and remove DNA/RNA. The dried samples were then dissolved in tetraethylammonium bromide, reduced with tris(2-carboxyethyl)phosphine and alkylated with iodoacetamide. Each sample was then acidified and loaded onto S-Trap cartridges (Protifi, NY) following the manufacturer's instructions. Retained proteins were digested with trypsin.

Peptides released from the cartridge were frozen and dried, reconstituted and loaded onto a liquid chromatography–mass spectrometer. Peptides were separated with a 70 min non-linear gradient (3-40% B, 0.1% formic acid (Line A) and 80% acetonitrile, 0.1% formic acid (LineB)) using a UltiMate 3000 RSLCnano high-performance liquid chromatographer (Thermo Fisher, MA). Samples were first loaded/desalted onto a 300μm x 5mm C18 PepMap C18 trap cartridge in 0.1% formic acid at 10 µL/min for 3 min and then further separated on a 75µmx50cm C18 column (EasySpray -C18 2 µm) with integrated emitter at 250nl/min. The eluent was directed to an Orbitrap Exploris 480 mass spectrometer (Thermo Fisher, MA) through the EasySpray source at a temperature of 320˚C, spray voltage 1,900V. Orbitrap full scan resolution was 60,000, re-imagined focus lens 50%, normalised ACG Target 300%. Precursors for tandem mass spectrometry were selected via a top 20 method. Intensity threshold 5.0 e3, charge state 2-7 and dynamic exclusion after 1 times for 35 seconds 10 parts per million (ppm) mass tolerance. ddMS2 scans were performed at 15,000 resolution, higher-energy collisional dissociation energy 27%, first mass 110 m/z, automatic gain control target standard. The acquired data were searched against the human proteome sequence database (https://www.uniprot.org/uniprot/) concatenated to the Common Repository for Adventitious Proteins v.2012.01.01 (ftp://ftp.thegpm.org/fasta/cRAP), using MaxQuant v1.6.43 (https://maxquant.net/perseus/). Parameters used: cysteine alkylation: iodoacetamide, digestion enzyme: trypsin, Parent Mass Error of 5ppm, fragment mass error of 10ppm. The confidence cut-off representative to false discovery rate (FDR)<0.01 was applied to the search result file. The report was generated to include protein groups, then further processed to exclude reversed sequences, common contaminants, proteins with only <2 unique peptides. Each protein was required to have more than 50% valid values (protein intensity>0) in at least one experimental group. Low quality samples were excluded from the analysis based on visual assessment of the spectral data and minimum number valid quantifiable proteins (at least 900

required). Protein intensity values were normalized by Log2 transformation, followed by median subtraction within each sample to account for unequal loading. The remaining missing values were then imputed by random selection from the left tail of the valid values distribution (1.8 standard deviations from the mean, within a 0.3 standard deviation range). T-test was used to identify proteins that were significantly different between groups. Permutation based FDR was calculated to account for multiple comparisons (250 randomisations) and FDR<0.05 was used as a threshold. All the analysis and visualisation of the proteomic data were conducted using Perseus 1.6.15.0.

The most significantly differentially abundant proteins (top 250 most abundant and 250 least abundant) between the control and long-term tracheostomised patients generated by this analysis were uploaded to Ingenuity® Pathway Analysis (IPA®) software, Qiagen (Hilden, Germany). IPA® uses a knowledge base derived from the scientific literature to relate genes or proteins based on their interactions and functions. Based on the uploaded dataset, the program algorithmically generates biological networks and functions and is able to assign activation (z-scores), where positive predicts activation and negative inhibition of a gene/pathway.

Metabolome testing

Non-targeted metabolomics was performed by Metabolon (Morrisville, NC). Briefly, recovery standards (quality control) were added to tracheal aspirates and proteins precipitated with methanol followed by centrifugation. The resulting extract was divided into five fractions: two (early and late eluting compounds) for analysis by ultra-high-performance liquid chromatography–tandem mass spectrometry (UHPLC-MS/MS) (positive ionisation), one for

analysis by UHPLC-MS/MS (negative ionisation), one for the UHPLC-MS/MS polar platform (negative ionisation), and one sample was reserved for backup. All methods used a Waters ACQUITY UHPLC (Milford, MA) and a Thermo Scientific Q-Exactive (Waltham, MA) high resolution/accurate MS interfaced with a heated electrospray ionisation (HESI-II) source and Orbitrap mass analyser operated at 35,000 mass resolution.

Metabolites were identified by automated comparison of the ion features in the experimental samples to a reference library of chemical standard entries that included retention time, molecular weight (m/z), preferred adducts, and in-source fragments as well as associated MS spectra. The metabolites were curated by visual inspection for quality control using software developed at Metabolon. Identification of known chemical entities is based on comparison to metabolomic library entries of commercially available purified standards. Xenobiotics were excluded from downstream analysis. Significant metabolites were determined using Welch's two-tailed t-test to identify biochemicals that differed significantly between two experimental groups. Analysis by one-way ANOVA with repeat measures identified biochemicals exhibiting significant changes in the serial sample analysis. An estimate of the FDR was calculated to take into account the multiple comparisons that normally occur in metabolomic-based studies.

Bacterial identification in airway samples

Nucleic acid extraction from tracheal aspirates was carried out using a PowerSoil DNA Isolation Kit (Qiagen, Hilden, Germany) in accordance with the manufacturer’s instructions. Bacterial profiling used the 16S rRNA gene targeting variable region four based on the Schloss wet-lab MiSeq standard operating procedure (SOP) by the NU-OMICS DNA Sequencing Research Facility (Northumbria University, UK). Resulting raw fastq data were processed using Mothur (version 1.45.2). Paired reads were merged and processed to remove sequences

containing ambiguous bases, homopolymers >8bp and sequences with a length >275bp. Sequences were aligned to the SILVA database and chimeras were removed using vsearch (v2.16.0). The remaining sequences were classified using the RDP database and sequences not identified as bacterial were removed from the downstream analysis. Samples yielding <4,000 reads were excluded. Reads were normalised by subsampling to 4,129 reads per sample. Analysis and visualisation of microbiome communities were conducted in R using the phyloseq package. Mann-Whitney U test was used for two variables and Kruskal-Wallis test for greater than two variables. FDR was applied to alpha diversity metrics where multiple tests were applied between groups. Bray-Curtis dissimilarity was calculated and explored by Principal Coordinates Analysis and differences between groups tested using PERMANOVA. Analysis of differentially abundant bacteria between groups was performed on taxa with >0.1% abundance across the cohort with FDR applied.

Biofilm assessment

Tracheostomy tubes were collected at the time of first tube change. The tube was cut to demonstrate the inner lumen of the mid-point of the tube, and subsequently fixed by treatment with glutaraldehyde. This was followed by dehydration through graded alcohols, mounting and gold coating. Specimens were examined for biofilm formation by an experienced operator using a Tescan Vega LMU Scanning Electron Microscope with digital image capture.

SUPPLEMENTARY TABLES

Table S1. Extended clinical details of the serial tracheostomy cohort.

Primary indication for tracheostomy	History of recurrent aspiration	Period of intubation prior to tracheostomy (days)	Ventilated at time of tracheostomy	Clinical tracheal aspirates microbiology while intubated (prior to tracheostomy)
Long-term ventilation	Yes	10	Yes	*Pseudomonas aeruginosa,* Serratia species
Long-term ventilation	No	11	Yes	None
Long-term ventilation	Yes	134	Yes	*Pseudomonas aeruginosa*
Long-term ventilation	No	142	Yes	*Pseudomonas aeruginosa, Staphylococcus aureus*
Upper airway obstruction	No	7	Yes	*Staphylococcus aureus, Stenotrophomonas maltophilia*
Upper airway obstruction	No	21	Yes	None
Long-term ventilation	No	26	Yes	*Staphylococcus aureus*
Long-term ventilation	No	24	Yes	None
Long-term ventilation	No	36	Yes	None

Table S2. Extended clinical details of the long-term tracheostomy cohort.

Primary indication for tracheostomy	Tracheostomy in situ (months)	History of recurrent aspiration	Currently ventilated	Clinical tracheostomy aspirate microbiology (within the last 12 months)
Long-term ventilation	18	Yes	No	*Klebsiella pneumoniae*, *Stenotrophomonas maltophilia*
Upper airway obstruction	35	Yes	No	*Stenotrophomonas maltophilia*, *Haemophilus influenzae*, Rhinovirus
Long-term ventilation	46	Yes	Yes	*Staphylococcus aureus, Streptococcus pneumoniae, Stenotrophomonas maltophilia, Serratia marcescens*, Respiratory syncytial virus, Rhinovirus
Upper airway obstruction	31	No	Yes	Influenza
Upper airway obstruction	26	No	No	*Stenotrophomonas maltophilia*, *Moraxella catarrhalis*, Acinetobacter species, *Delftia acidovorans*

Long-term ventilation	9	Yes	Yes	*Pseudomonas aeruginosa*, *Streptococcus pneumoniae*, *Escherichia coli*, Adenovirus
Upper airway obstruction	140	Yes	Yes	*Staphylococcus aureus*
Upper airway obstruction	35	Yes	No	No growth
Long-term ventilation	34	No	Yes	*Pseudomonas aeruginosa*, *Haemophilus influenzae*, *Stenotrophomonas maltophilia*, *Serratia marcescens*, *Moraxella catarrhalis*, Adenovirus
Long-term ventilation	47	Yes	Yes	*Haemophilus influenzae*
Upper airway obstruction	13	Yes	No	*Haemophilus influenzae, Stenotrophomonas maltophilia, Moraxella catarrhalis, Enterobacter cloacae*
Long-term ventilation	7	Yes	Yes	*Pseudomonas aeruginosa, Klebsiella pneumoniae, Citrobacter freundii complex*

Upper airway obstruction	37	Yes	No	*Pseudomonas aeruginosa*
Long-term ventilation	7	No	Yes	No growth
Long-term ventilation	67	Yes	Yes	*Pseudomonas aeruginosa, Staphylococcus aureus,* Serratia species*,* Rhinovirus
Long-term ventilation	76	Yes	No	*Staphylococcus aureus*
Long-term ventilation	51	No	No	No growth
Long-term ventilation	28	No	Yes	*Moraxella catarrhalis*, Acinetobacter species
Upper airway obstruction	31	No	Yes	No growth
Long-term ventilation	25	Yes	Yes	*Staphylococcus aureus, Stenotrophomonas maltophilia,* Rhinovirus
Long-term ventilation	21	Yes	No	*Pseudomonas aeruginosa, Staphylococcus aureus, Stenotrophomonas maltophilia*

Upper airway obstruction	10	Yes	No	*Pseudomonas aeruginosa, Staphylococcus aureus, Streptococcus pneumoniae, Klebsiella pneumoniae, Delftia species, Escherichia coli,* Rhinovirus
Upper airway obstruction	35	No	No	*Haemophilus influenzae*, *Pseudomonas aeruginosa*
Long-term ventilation	11	No	No	*Haemophilus influenzae*, *Pseudomonas aeruginosa*, *Staphylococcus aureus*

Table S3. Extended clinical details of the control cohort.

Bronchoscopy indication/findings	Additional significant co-morbidities
Laryngomalacia	None
Laryngomalacia, unilateral vocal cord palsy	Coarctation aorta repair
Pharyngolaryngomalacia, tracheobronchomalacia	None
Obstructive sleep apnoea	Achondroplasia
Obstructive sleep apnoea	Global developmental delay
Obstructive sleep apnoea, choking episodes	None
Recurrent croup	Chronic lung disease of prematurity
Obstructive sleep apnoea	None
Laryngomalacia	None
Obstructive sleep apnoea	None
Pharyngomalacia	None
Chocking episodes	None
Obstructive sleep apnoea	None

Table S4. Metabolomic dipeptide sub-pathway findings from tracheal aspirates. Metabolite fold change is demonstrated in the post-tracheostomy group relative to the time of tracheostomy. Significant values (p<0.05) are in bold and marked with an asterisk. Statistical analysis was by One-Way Repeated Measure ANOVA: time of tracheostomy n=9, 1 week n=9, 1 month n=6, 3 months n=5.

**Biochemical Name**	**Post-tracheostomy**		
	**1 week**	**1 month**	**3 months**
alanylleucine	**7.20***	2.85	**5.92***
cyclo(leu-pro)	**3.01***	2.48	1.68
glycylisoleucine	**3.88***	1.66	2.00
glycylleucine	**5.38***	1.31	1.69
glycylvaline	**10.31***	1.96	2.55
histidylalanine	**2.19***	1.20	1.28
isoleucylglycine	**3.04***	1.34	1.18
leucylalanine	**10.83***	3.45	**6.93***
leucylglycine	**12.07***	5.38	**9.18***
lysylleucine	**2.92***	1.06	1.23
phenylalanylalanine	**4.58***	1.48	2.94
phenylalanylglycine	**2.59***	1.36	1.65
threonylphenylalanine	**6.00***	1.64	2.95
tyrosylglycine	**1.25***	0.98	1.04
valylglycine	**2.82***	1.24	1.26
valylleucine	**8.27***	2.31	4.98
leucylglutamine	**3.41***	1.61	1.85

SUPPLEMENTARY FIGURES

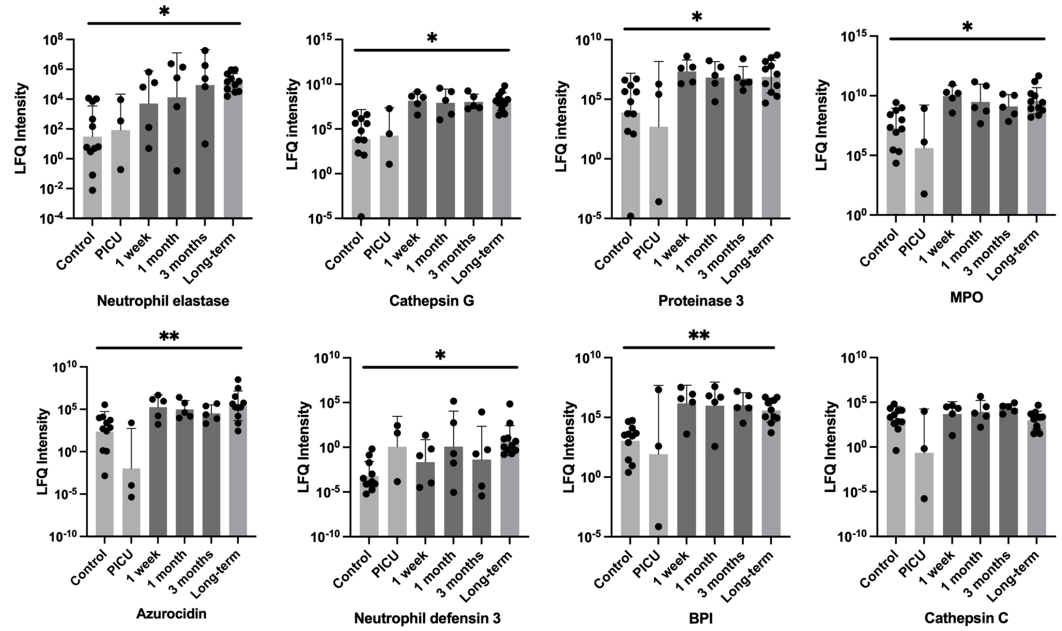

Figure S1 Individual serial protein abundance in tracheal aspirates for neutrophil primary granules. Significant differences between controls and long-term tracheostomies are demonstrated. No other statistically significant differences were identified comparing between groups. Upper aspect of each column indicates the mean and the whisker the standard deviation. Time of tracheostomy (in the paediatric intensive care unit (PICU)) to three months post-procedure is shown. Controls and long-term tracheostomies are also demonstrated. Statistical analysis was by two-sample t-Test, *p<0.05, **p<0.01. Controls n=11, serial; PICU n=3, 1 week n=5, 1 month n=5, 3 months n=5, long-term tracheostomy (>6 months) n=11. LFQ, Label-free quantification; BPI, bactericidal permeability-increasing protein; MPO, myeloperoxidase.

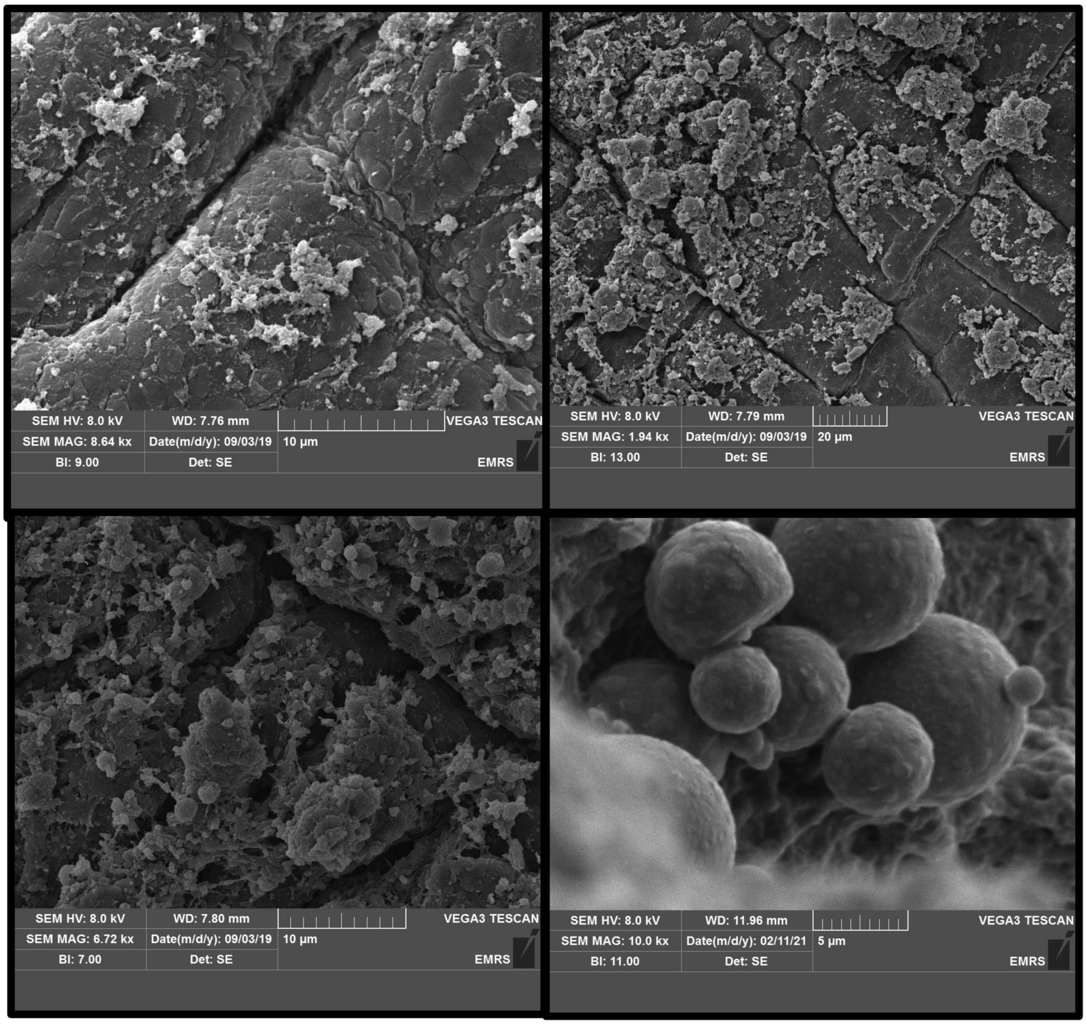

Figure S2 Biofilms are frequently found in tracheostomy tubes as early as one week post-procedure. Representative (n=9) scanning electron microscopy images of biofilms on the inner lumen of paediatric tracheostomy tubes at one-week post placement.

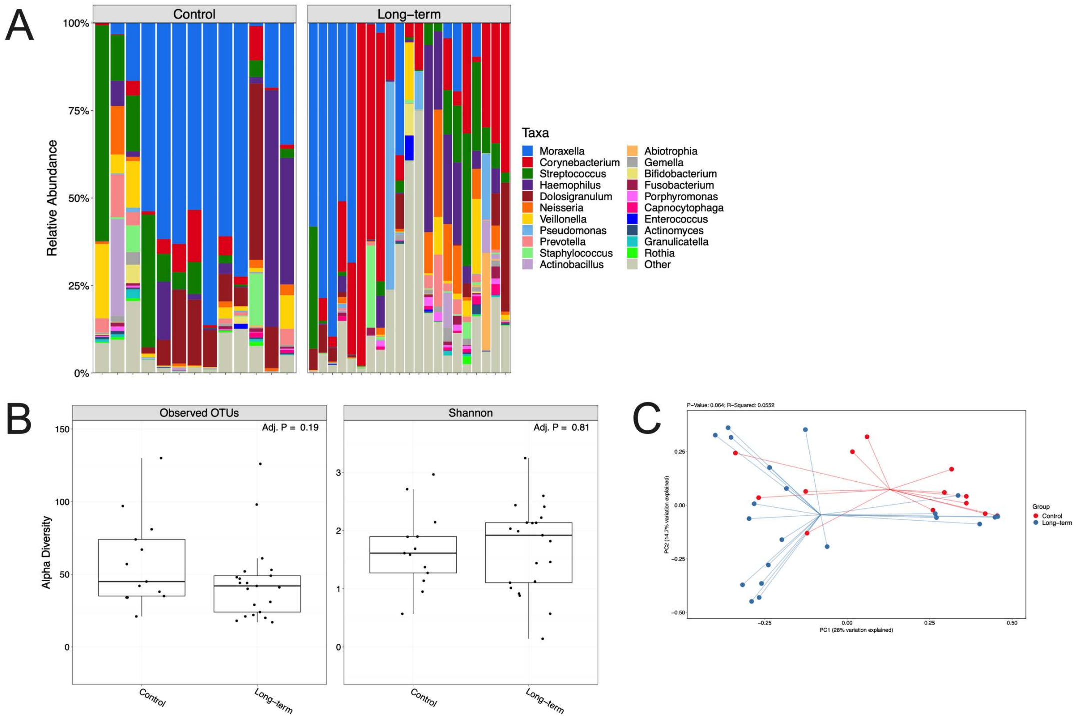

Figure S3 Nose swabs from long-term tracheostomised children demonstrate a similar abundance and diversity of organisms compared to controls. *(A)* 16S rRNA sequencing demonstrating hierarchical clustering of nose swab samples by bacterial relative abundance at observed operational taxonomic unit (OTU) level where each column represents a patient's sample. *(B)* OTUs and Shannon alpha diversity and *(C)* principal component analysis for nasal swabs. Long-term tracheostomy n=21, controls n=13.
